# The Indirect Effects of Self-Efficacy on Cannabis Use via Cannabis Protective Strategies: A Preliminary Study

**DOI:** 10.26828/cannabis.2018.02.007

**Published:** 2018-07-07

**Authors:** Eleftherios M. Hetelekides, Alexander J. Tyskiewicz, Mark A. Prince, R. Lorraine Collins

**Affiliations:** 1 Research Institute on Addictions, University at Buffalo, Buffalo, NY, USA; 2 Department of Psychology, Colorado State University, Fort Collins, CO, USA; 3 Department of Community Health and Health Behavior, University at Buffalo, Buffalo, NY, USA

**Keywords:** self-efficacy, cannabis use, marijuana, cannabis protective strategies

## Abstract

Cannabis use has more than doubled in the past decade and nearly three of 10 users develop a cannabis use disorder. The increase in users, combined with the ongoing changes in the medical, legal and social status of cannabis, has contributed to interest in the antecedents of cannabis use. In the current preliminary study, we gathered information from a community sample (N = 54) of regular cannabis using young adults. Assessments included perceived self-efficacy for reducing intake or abstaining (SE-R/A), use of cannabis protective strategies (CPS), and average quantity of cannabis use per day. We systematically explored which specific CPS were most strongly associated with self-efficacy and cannabis use. Three clinically relevant subgroups of CPS emerged from this analysis: strategies strongly associated with only self-efficacy, only cannabis use, and both variables. We hypothesized that self-efficacy would be associated with less cannabis use via use of CPS. Among specific CPS examined, “Use a little and then wait to see how you feel before using more” had the strongest association with self-efficacy while “Avoid methods of using cannabis that can make you more intoxicated than you would like” had the strongest negative association with cannabis use. We observed a significant indirect relationship from self-efficacy to cannabis use through use of CPS. Our findings suggest that use of CPS is a potential mechanism by which cannabis users reduce use, and a more proximal antecedent of cannabis use than personal confidence in one’s ability to stop using. These preliminary findings highlight the value of studying individual strategies. They also may have implications for promoting use of cannabis protective strategies when treating those with cannabis use problems.

In the US, the legal status of cannabis continues to evolve. As of 2017, 29 states have legalized the use of medical cannabis, and nine permit recreational use. Nationwide, cannabis is the most widely used illicit drug and overall usage rates are increasing (Johnston, O’Malley, Bachman, Schulenberg, & Miech, 2015). As the population of users has increased, the number of individuals diagnosed with Cannabis Use Disorder (CUD) has followed suit (Hasin, 2018). Young adults are at particularly high levels of risk for abusing cannabis and developing associated negative consequences (Pedersen, Hummer, Rinker, Traylor, & Neighbors, 2016). In fact, research has shown that (a) daily use among college students is at its highest level in over three decades, (b) 1 in 10 non-college attending young adults report daily use, and (c) about 1 in 17 high school seniors report using cannabis daily (Johnston et al., 2015). A variety of negative consequences may follow from excessive use. These include CUD; motor vehicle accidents; decreased academic performance; impaired respiratory and cardiovascular health; unintentional childhood exposure; as well as higher risk for experiencing psychiatric symptoms (Hasin, 2018; National Academies of Sciences, Engineering, and Medicine, 2017).

Psychosocial interventions are generally used to help individuals to lessen their cannabis use. Cognitive Behavioral Therapy (CBT) and Motivational Enhancement Therapy (MET) are two intervention approaches that have been significantly more effective in reducing cannabis use and consequences compared to control conditions (Copeland, Swift, Roffman, & Stephens, 2001; Walker et al., 2011). Even so, the efficacy of these treatments could be improved and new psychosocial interventions could be developed. To achieve this, it is essential to understand the mechanisms of change involved in reducing cannabis use. Therapeutic methods such as CBT include the use of behavioral strategies that promote abstinence or decreased drug use and related consequences (Copeland et al., 2001). Although such interventions are theoretically supported (LaBrie, Napper, Grimaldi, Kenney, & Lac, 2015), clinical success has been limited and research examining treatments that directly target individually-tailored Protective Behavioral Strategies (PBS) for substance use are rare, despite having shown promise (Bingham et al., 2011). Similarly, examination of the role of self-efficacy is lacking, even in research that prioritizes its enhancement as a means of reducing substance use and consequences (Litt, Kadden, & Petry, 2013). This is in part due to a lack of concrete procedures for increasing self-efficacy levels (Kadden & Litt, 2011).

Self-efficacy is an important construct in the behavior change literature, especially with regard to substance abuse disorders. Bandura (1977) first described the concept as one’s belief in one’s personal ability to effectively execute the behaviors required to produce a specific outcome. Self-efficacy has strong explanatory power in predicting and mediating treatment effects across a variety of addictive behaviors (DiClemente, Prochaska, & Gibertini, 1985; Litt, Kadden, & Dabela, 2008). It has been shown to play a positive role in treatment for alcohol use disorders (Adamson, Sellman, & Frampton, 2009; Maisto et al., 2015), smoking cessation (Condiotte & Lichenstein, 1981; DiClemente, Fairhurst, & Piotrowski, 1995; Gwaltney, Metrik, Kahler, & Shiffman, 2009) and outcomes of cannabis treatment (Litt & Kadden, 2015; Litt et al., 2008; Litt et al., 2013; Stephens, Wertz, & Roffman, 1995).

Protective behavioral strategies have been identified as a mechanism for reducing harm related to managing risky behaviors, including substance use (Pedersen et al., 2016). The alcohol research literature has provided evidence of negative associations between frequency of PBS use and alcohol use, and/or alcohol-related negative consequences, across a variety of groups (Borden et al., 2011; Braitman, Linden-Carmichael, & Henson, 2017; Kenney, Napper, LaBrie, & Martens, 2014; LaBrie, Kenney, & Lac, 2010). Additional findings indicate that PBS use plays a moderating role in the relationship between binge drinking and alcohol problems, and that interventions focusing on increasing the number of strategies used by individuals within risky situations may be beneficial for reducing problematic alcohol-related behaviors and consequences (Borden et al., 2011; Braitman et al., 2017). The fledgling literature on the effects of PBS on cannabis use yields similar evidence. The results of research that has examined the associations among use of protective strategies, cannabis use, and negative consequences indicate that PBS use for cannabis is inversely associated with both other variables (e.g., Bravo, Prince, Pearson, & Marijuana Outcomes Study Team, 2017; Pedersen et al., 2016). Further, use of protective strategies may atemporally (without implying causation; see Winer et al., 2016) mediate the relationship between self-efficacy and cannabis use/consequences, with PBS use serving as a mechanism by which self-efficacy is manifested as behavioral control.

In the current study, we examined whether the use of cannabis protective strategies (CPS) may atemporally mediate the relationship between self-efficacy to reduce or abstain (SE-R/A) from using cannabis, and cannabis use (Winer et al., 2016). CPS are behaviors that occur more proximally to cannabis use relative to self-efficacy, which is expected to motivate use of such strategies. We examined the relations among average cannabis quantity per day (i.e., the sum of all joints reported per day of the week divided by 7), use of CPS, and participant’s SE-R/A from cannabis use, and hypothesized that greater levels of SE-R/A would be negatively associated with quantity of cannabis use and positively associated with the use of CPS. Moreover, we hypothesized that particularly effective CPS could be identified across participants. This item-level analysis provided a deeper, qualitative look at the relations among SE-R/A, use of CPS, and cannabis use. It also could provide clinically relevant insights into the types of strategies that may be more or less beneficial to individuals across the range of SE-R/A.

## METHOD

### Participants

Participants were community residents (*N* = 54; 34 (63%) men and 20 (37%) women) from the Buffalo, NY metropolitan area, who self-reported regular cannabis use. Although use of medical cannabis (mainly oil-based CBD products) now is legal in New York state, our participants were recreational users of cannabis and did not use cannabis for medicinal purposes. Selection criteria included: being 18-30 years of age (*M* = 24.33; *SD* = 3.07); possession of a high school diploma or GED equivalent, and using cannabis at least 3 times per week. Along with cannabis use, most participants (78%) reported social drinking (no more than 3 drinks per week; *M* = 1.11 drinks per day; *SD* = 1.02) and low rates of lifetime use of a variety of substances, including for tobacco products and cocaine. The majority of the sample was single (85.1%) and not attending school (83.3%). Some (22.2%) participants were unemployed and 58.5% reported a total household income of less than $20,000. The sample indicated they were European American (44.4%), African American (27.8%), from more than one background (16.7%), unknown (3.7%), and other (5.6%). The majority (74.1%) of participants identified as non-Hispanic.

We used Facebook advertising to recruit participants. Individuals who clicked the advertisement were taken to our study page and asked to call or leave a Facebook message with their phone number to indicate interest. Eligibility was identified through a 20-minute phone screen. Participants were found ineligible if they reported a history of serious or ongoing legal or medical issues, legal issues due to cannabis use, signs of severe mental illness, or drug abuse problems other than those associated with cannabis (e.g., abusing other illicit or licit drugs). This study was approved by the Institutional Review Board of the University at Buffalo. All participants provided their written informed consent, in which they agreed to be audio recorded. Each received $50 for participating in all aspects of the study.

### Procedure

Each participant was assigned to one of the eight semi-structured focus groups conducted for the study. Each group was facilitated by two trained clinicians. During each group, participants were prompted to identify reasons they could imagine someone might want to quit or “cut down” on cannabis and to describe strategies that they felt could be used to achieve this goal. At the end of the group discussion, they individually completed a computerized survey that assessed the constructs of interest, using the measures described below.

### Measures

#### *Marijuana Use Questionnaire* (MUQ; Collins, Vincent, Yu, Liu, & Epstein, 2014).

We used the MUQ to assess the typical quantity of cannabis used. Participants were shown an image of an “average-sized joint” (1/2 a gram) and instructed to report the typical number of average-sized joints or equivalent amount used based on mode of use (e.g., smoke, vaporize), for each day of the week over the past 30 days. On average, participants reported smoking 3.10 joints on any given day of the week (*SD* = 2.12, Range = 0.71 – 10.43). Two participants’ scores were extreme outliers, so they were winsorized to the 95th percentile.

#### *Protective Behavioral Strategies for Marijuana* (PBSM; Pedersen et al., 2016).

This measure was used to assess use of cannabis protective strategies. Participants indicate the degree to which they used a pre-defined list of behaviors to lessen cannabis use and related negative consequences, on a scale, from 1 = never to 6 = always. Sample items included “Use a vaporizer or other smokeless method to avoid carcinogens”, “Avoid buying marijuana”, and “Avoid using marijuana early in the day”. In the current study, we used the 50 items in the original PBSM, rather than the 39-items in the measure finalized by Pedersen et al. Because the examination of strategy use is in its infancy and the PBSM is a relatively new measure, we felt that the additional 11 items might provide useful insights given our research goals. The 11 items that were not included in the original 39-item single factor PBSM have been italicized in Table 1. For this sample, individuals on average and across all items reported using strategies “occasionally” (*M* = 2.94, *SD* = 1.00).

#### *Self-Efficacy to Reduce Consumption or Abstain from Using Cannabis* (SE-R/A; Stephens et al., 1995).

This measure asked participants to rate how confident they would be in resisting the temptation to smoke cannabis in the context of 20 different situations. Responses were recorded on a 7-point scale from 1 (not at all confident) to 7 (extremely confident). Examples include asking participants how confident they would be in reducing or abstaining if “Offered marijuana by someone”, “Bored with nothing to do”, and “Stressed out and needing to calm down.” Stephens et al. examined the internal consistency of the SE-R/A at pretreatment and posttreatment, and reported alphas of 0.89 and 0.94, respectively. For the current sample, participants rated their SE-R/A levels at 3.54 on average (*SD* = 1.82), and alpha reliability was excellent (α = 0.96).

### Analysis Plan

We tested our atemporal mediation hypothesis that cannabis use is predicted by SE-R/A indirectly, via use of CPS, using a path analysis (see model in Figure 1). Cannabis use is typically a highly skewed count variable; however, in the present study of regular to heavy cannabis users, the cannabis use variable was best modeled as approximately normal. Analyses were conducted using M*plus* 7.4 (Muthén & Muthén, 1998–2012). The a priori path model is fully saturated, which precludes tests of overall model fit.

The primary challenge in making appropriate determinations regarding the strength of an indirect effect is that the product of two regression slopes is not normally distributed. The violation of the normality assumption results in a loss of statistical power for many traditional approaches to testing mediation (e.g., the Sobel Test). In order to circumvent this issue, the best practices approach is to assess asymmetrical confidence intervals (ACIs) that best represent the true distribution of the product of coefficients. ACIs that do not contain zero are considered to be statistically significant. We examined the indirect effect of self-efficacy on outcomes using bias-corrected bootstrapped estimates (Efron & Tibshirani, 1993) based on 10,000 bootstrapped samples, which provides a powerful test of mediation (Fritz & MacKinnon, 2007) and are asymmetrical. Statistical significance was determined by 95% bias-corrected bootstrapped confidence intervals that do not contain zero.

We used Pearson’s correlations to explore item-level analyses of CPS associations with self-efficacy and cannabis use. The goal of these analyses was to assess the degree of variability in the magnitude of the associations between each individual protective strategy (based on PBSM items) with self-efficacy and cannabis use to determine which specific strategies were most strongly linked to each variable.

## RESULTS

### Typical Cannabis Use

The current sample used cannabis near daily, reporting on average 27.02 (*SD* = 6.50) using days in the past month and 6.71 (*SD* = 1.06) in the week on average. Mean age of first use was 14.65 (*SD* = 2.91), and participants reported starting to use cannabis regularly on average at age 16.89 (*SD* = 3.18). Regarding quantity consumed, participants reported an average of 75.46 (*SD* = 47.96) standard joints (1/2 a gram) in the past month, which is equivalent to 1.50 (*SD* = 0.94) grams per using day. The sample also reported experiencing an average of 8.58 (*SD* = 5.62) out of 27 cannabis-related problems in the past month. Subjective intoxication was rated on average at 6.70 (*SD* = 1.77) out of 10.

### Prediction of Cannabis Use by Self-Efficacy

We used path analysis to examine whether cannabis use was predicted by self-efficacy (as measured by the SE-R/A) indirectly, via CPS use (see Figure 1). We found that our measure of self-efficacy explained 23% of the variance in CPS, as measured by the PBSM. Self-efficacy and CPS use explained 25.5% of the variance in cannabis use. Self-efficacy significantly and positively predicted CPS use (*b* = .27, *SE* = .09, *p* = .004, 95% bias-corrected bootstrapped CI [.14, .72], β = .48), but did not significantly predict cannabis use (*b* = −.13, *SE* = .12, *p* = .27, 95% bias-corrected bootstrapped CI [−.35, .09], β = −.16). However, in bivariate models, Self-efficacy significantly negatively predicted cannabis use. Use of CPS significantly negatively predicted cannabis use (*b* = −.56, *SE* = .22, *p* = .01, 95% bias-corrected bootstrapped CI [−.96, −.10], β = −.41). The indirect effect of self-efficacy predicting cannabis use via CPS use was statistically significant (*b* = −.15, *SE* = .08, *p* = .03, 95% bias-corrected bootstrapped CI [−.34, −.05], β = −.19).

### Associations among Use of Cannabis Protective Strategies, Self-Efficacy, and Typical Cannabis Use

We sought to identify specific cannabis protective strategies that were particularly strongly associated with greater levels of self-efficacy (i.e., the SE-R/A) and less cannabis use, across participants. We identified three broad patterns of findings based on medium to large effect sizes (see Table 1). Specifically, we found: 1) a set of six strategies with medium to large effect sizes associated with self-efficacy, but not with typical quantity of cannabis; 2) a set of four strategies with medium to large effect sizes associated with typical quantity of cannabis use, but not with self-efficacy; and, 3) a set of 19 strategies with medium to large effect sizes associated with both self-efficacy and typical quantity of cannabis used. Overall, 29 items had medium to large effects related to at least one variable, including four items that did not load onto the original 39-item PBSM, identified by Pedersen et al. (2016).

Some studies in the alcohol literature have demonstrated that PBS can be differentiated, and that different types of PBS are more effective for certain individuals (e.g., Linden, Kite, Braitman, & Henson, 2014). Table 1 includes all 50 strategies from the original PBSM measure (Pedersen et al., 2016), presented in descending order based on effect sizes and grouped into four clusters. The first cluster consists of strategies with medium to large effect sizes in relation to self-efficacy (i.e., SE-R/A), then medium to large effect sizes with cannabis use, then medium to large effect sizes with both self-efficacy and cannabis use. The final set of strategies had less than medium sized effects.

## DISCUSSION

In the present study, we examined the role of cannabis protective strategies (CPS) in atemporally mediating the effects of reported self-efficacy to reduce or abstain (measured by the SE-R/A) on typical quantity of cannabis used. Specifically, higher levels of self-efficacy were significantly associated with more frequent strategy use. More frequent use of CPS was in turn significantly associated with lower levels of cannabis use. Some subsets of strategies had stronger effect sizes in relation to greater self-efficacy and/or less cannabis use. Our findings are framed using the distinction made between temporal and atemporal mediation (Winer et al., 2016). Atemporal analyses do not speak to the causality of why mediation is occurring as a function of time. In this way, the path model is not implying a one directional relationship between the variables. Therefore it is possible and theoretically likely that in reality this relationship is reciprocal, for example, less cannabis use leading to more use of CPS, and greater levels of self-efficacy.

We grouped the strategies, assessed using items from the PBSM, based on the size of the effect related to self-efficacy and cannabis use. The first group of strategies can be broadly classified as strategies used to modify cannabis use in specific ways, and are highly associated with SE-R/A, but not cannabis use. Two PBSM items that did not load onto the original factor described by Pedersen et al. (2016) were a part of this group (“Use higher potency marijuana so you can take less hits and avoid lung damage” and “Use your own marijuana (if alone or sharing with friends) so you know what you are using”). Efforts to modify cannabis use are thus linked to SE-R/A. However, effective use of CPS (i.e., use of strategies that leads to less cannabis use) does not necessarily follow attempts to use these strategies. For example, “Do not keep marijuana in the car, whether as a driver or passenger” is a strategy that even if successfully employed to reduce the risk of consequences, does not have direct implications for overall cannabis use. These modification-based strategies may still play a significant role in reducing the number of negative, cannabis related consequences experienced by individuals. The four CPS in the next group were strongly associated with reductions in cannabis use but not SE-R/A beliefs. Examples include “Avoid mixing marijuana with other drugs”, “Stop using marijuana if you become anxious or paranoid”, and “Avoid driving a car after using.” These strategies may be a particularly useful subset for individuals seeking to decrease their cannabis use, because they can be used even by individuals who have low self-efficacy (as measured by the SE-R/A). This maybe because each of these strategies operate within a context where there are a priori reasons that provide situational motivation to limit use, such as feeling anxious or needing to operate a motor vehicle. The strategies from the next cluster are highly associated with both greater self-efficacy and less typical cannabis use. Two of these strategies also did not load onto the original PBSM factor structure (specifically, “If attending a party or going out to a social event (e.g., bar), decide in advance whether you want to use marijuana or not” and “Only use before special events (e.g., movies, concerts) or on special occasions”). Additional examples from this cluster of strategies (and from the original 39-item PBSM) include “Avoid using marijuana habitually (that is, everyday or multiple times a week)”, “Avoid using marijuana early in the day”, and “Take periodic breaks if it feels like you are using marijuana too frequently.” These strategies modified cannabis use behaviors and provided concrete reductions in overall use. These strategies may be useful for individuals who would most benefit from bolstered levels of self-efficacy, because using them likely will result in reaching one’s goals, thereby creating a positive cycle that boosts self-efficacy. Together, the item-level analysis of strategies listed in the PBSM provides qualitative support for the conceptual framework of the path model, by identifying the strategies that are most strongly associated with self-efficacy, with typical cannabis use, and with both. The item-level analysis in tandem with the indirect effects provide evidence that use of CPS may be a mechanism by which self-efficacy contributes to reductions in cannabis use.

Cannabis-specific treatment programs typically are designed to provide cannabis users with useful, viable tools for regulating their use and reducing harm. In this study, the use of CPS had a strong cross-sectional association with lower levels of cannabis use. Moreover, we found that CPS use had a more proximal association to cannabis use than self-efficacy, as measured by the SE-R/A. Thus, we recommend that use of CPS serve as high priority targets for promoting abstinence and/or reductions in cannabis use. Further, we recommend that interventionists work to bolster self-efficacy, as it may increase the number of strategies that cannabis users employ as well as being directly related to lessening cannabis use. Additionally, because strong self-efficacy beliefs are associated with generally positive outcomes after treatment, encouraging strategy use that naturally promotes increased levels of self-efficacy may help to maximize positive treatment outcomes. In sum, the relationship between these variables may be most effectively utilized in clinical settings by boosting the existing positive feedback loop to minimize recurrent cannabis use and problems. Finally, this research presents preliminary evidence that highlights the clinical value of studying different types of strategies, and encourages additional research to examine how different types of CPS may be more or less useful to different individuals.

There are several strengths and limitations to this study. First, we collected these data from a community sample of young-adults who reported regular use of cannabis - a population at high risk for experiencing negative cannabis-related consequences. It is also the first study to examine the relationship among self-efficacy, use of CPS, and cannabis use, and which provides both aggregate and item-level analyses of these relationships. Limitations include the small sample size, lack of information gathered regarding participant’s use of THC concentrates, as well as the correlational design of the study.

The use of CPS has been identified as a broad negative predictor for cannabis use quantity, frequency, and consequences. Our findings are consistent with previous research in suggesting that intervention programs should promote strategy use as a way of helping individuals attenuate their use and level of risk (Bravo, Anthenien, Prince, Pearson, & Marijuana Outcomes Study Team, 2017; Pedersen et al., 2016). CPS also have been shown to buffer and enhance a variety of risk and protective factors (Bravo, Anthenien et al., 2017). Though more research is needed to identify specifically what other variables may be involved in the relationships examined in this manuscript, this study suggests that use of protective strategies are one mechanism by which self-efficacy is associated with reductions in cannabis use.

## Figures and Tables

**Table 1.** Pearson’s r effect sizes among PBSM items, self-efficacy to reduce/abstain (SE-R/A) and cannabis use (CU).

**Strategies with Medium to Large Effects with SE-R/A Only (bolded)**		

PBSM Item	SE-R/A	CU

*Use higher potency marijuana so you can take less hits and avoid lung damage.*	**.483**	−.221
Avoid using marijuana for several days in advance of a big test, interview, performance, or other engagement for which you need to be crisp and are being evaluated.	**.436**	−.165
Do not keep marijuana in the car, whether as a driver or passenger.	**.408**	−.129
*Use your own marijuana (if alone or sharing with friends) so you know what you are using.*	**.387**	−.237
Keep track of your costs to get an accurate picture of how much you spend on marijuana.	**.344**	−.171
To decrease tolerance, take a break for a week or two, or take longer breaks than usual between use.	**.313**	−.271

**Strategies with Medium to Large Effects with Cannabis Use Only (bolded)**		

PBSM Item	SE-R/A	CU

Avoid using marijuana before work or school.	.289	**−.412**
Stop using marijuana if you become anxious or paranoid.	.112	**−.360**
Avoid mixing marijuana with other drugs.	.159	**−.358**
Take a break from using if feeling a loss of motivation.	.246	**−.320**

**Strategies with Medium to Large Effects with SE-R/A and Cannabis Use (bolded)**		

PBSM Item	SE-R/A	CU

Avoid using marijuana habitually (that is, every day or multiple times a week).	**.483**	**−.451**
Use a little and then wait to see how you feel before using more.	**.461**	**−.453**
Avoid methods of using marijuana that can make you more intoxicated than you would like (e.g., using large bongs, volcano, ‘edibles,’ etc.).	**.400**	**−.497**
*If attending a party or going out to a social event (e.g., bar), decide in advance whether you want to use marijuana or not.*	**.430**	**−.458**
*Only use before special events (e.g., movies, concerts) or on special occasions.*	**.397**	**−.460**
Avoid using marijuana before engaging in physical activity (i.e., exercise, hiking).	**.390**	**−.467**
Limit the amount of marijuana you smoke in one sitting.	**.455**	**−.397**
Use enough only to achieve a slight buzz or to avoid getting “too high”.	**.400**	**−.422**
Only use marijuana on private property.	**.456**	**−.352**
Take periodic breaks if it feels like you are using marijuana too frequently.	**.380**	**−.426**
Avoid buying marijuana.	**.440**	**−.365**
Avoid using marijuana early in the day.	**.395**	**−.399**
Avoid using marijuana out of boredom.	**.446**	**−.348**
Only use one time during a day/night.	**.396**	**−.388**
Pass on shared joints, bongs, etc. if already feeling high.	**.303**	**−.429**
Avoid using when feeling anxious (e.g., using to calm you down or stop worrying).	**.327**	**−.404**
Avoid driving a car after using.	**.325**	**−.373**
Avoid using marijuana in concentrated forms (e.g., hashish, hashish/honey oil, kief, marijuana butter/oil, etc.) to avoid getting too high.	**.333**	**−.342**
Only use when you know you have nothing important to do for the rest of the day/night.	**.312**	**−.308**

**Strategies with Less Than Medium Effects (bolded)**		

PBSM Item	SE-R/A	CU

*Use eye drops so others do not know you have used.*	.263	−.254
*Use a vaporizer or other smokeless method to avoid carcinogens.*	.258	−.229
Avoid using marijuana to cope with emotions such as sadness or depression.	.229	−.242
Avoid using marijuana in large gatherings or crowds.	.285	−.185
*Use only at home.*	.157	−.269
Avoid using marijuana in public places.	.120	−.289
Only use at night (that is, not during the day).	.236	−.166
Avoid using marijuana if currently taking any kind of prescription drug that might intensify the effects (e.g., make you feel more tired).	.144	−.219
Avoid possibilities of legal repercussions (e.g., smoke in a safe place like home, avoid having marijuana with you where you might get searched, etc.).	.152	−.206
*Only purchase marijuana from a trusted source.*	.174	−.139
Use a designated driver (i.e., someone who has not used) after using marijuana.	.109	.201
Limit, use to weekends.	.204	−.093
Excuse yourself from the room if people are smoking marijuana and you feel uncomfortable or do not wish to be offered marijuana.	.126	−.147
*Only use marijuana after completing all of the day’s responsibilities.*	.169	.093
*Avoid mixing marijuana with alcohol.*	.050	−.189
Avoid use while spending time with family.	.045	−.192
Buy less marijuana at a time so you smoke less.	.106	−.066
Avoid situations that you anticipate being pressured to use marijuana.	.131	−.035
Having a set amount of “times” you take a hit (e.g., passing on a shared joint if you have already hit that limit).	.155	−.010
*Use marijuana only among trusted peers.*	.048	−.089
Avoid bringing marijuana into events or venues where you are likely to be searched.	.064	−.031

*Note.* Italicized items were not included in the single factor PBSM scale developed by Pedersen et al., 2016.
